# Transpacific Transport of Asian Peroxyacetyl Nitrate (PAN) Observed from Satellite: Implications for Ozone

**DOI:** 10.1021/acs.est.4c01980

**Published:** 2024-05-22

**Authors:** Shixian Zhai, Daniel J. Jacob, Bruno Franco, Lieven Clarisse, Pierre Coheur, Viral Shah, Kelvin H. Bates, Haipeng Lin, Ruijun Dang, Melissa P. Sulprizio, L. Gregory Huey, Fred L. Moore, Daniel A. Jaffe, Hong Liao

**Affiliations:** † Earth and Environmental Sciences Programme and Graduation Division of Earth and Atmospheric Sciences, Faculty of Science, The Chinese University of Hong Kong, Sha Tin , Hong Kong SAR, China; ‡ John A. Paulson School of Engineering and Applied Sciences, Harvard University, Cambridge, Massachusetts 02138, United States; § Université libre de Bruxelles (ULB), Spectroscopy, Quantum Chemistry and Atmospheric Remote Sensing, Brussels B-1050, Belgium; ∥ NOAA Chemical Sciences Laboratory, Earth System Research Laboratories, & Cooperative Institute for Research in Environmental Sciences, University of Colorado, Boulder, Colorado 80305, United States; ⊥ School of Earth and Atmospheric Sciences, Georgia Institute of Technology, Atlanta, Georgia 30332, United States; # NOAA Global Monitoring Laboratory, Boulder, Colorado 80305, United States; ∇ Cooperative Institute for Research in Environmental Sciences, University of Colorado Boulder, Boulder, Colorado 80309, United States; ○ School of Science, Technology, Engineering, and Mathematics, University of Washington, Bothell, Washington 98011, United States; ◆ Department of Atmospheric Sciences, University of Washington, Seattle, Washington 98195, United States; ¶ Jiangsu Key Laboratory of Atmospheric Environment Monitoring and Pollution Control, Collaborative Innovation Center of Atmospheric Environment and Equipment Technology, School of Environmental Science and Engineering, Nanjing University of Information Science and Technology, Nanjing 210044, China

**Keywords:** peroxyacetyl nitrate, ozone, atmospheric chemistry modeling, satellite remote sensing, satellite retrieval

## Abstract

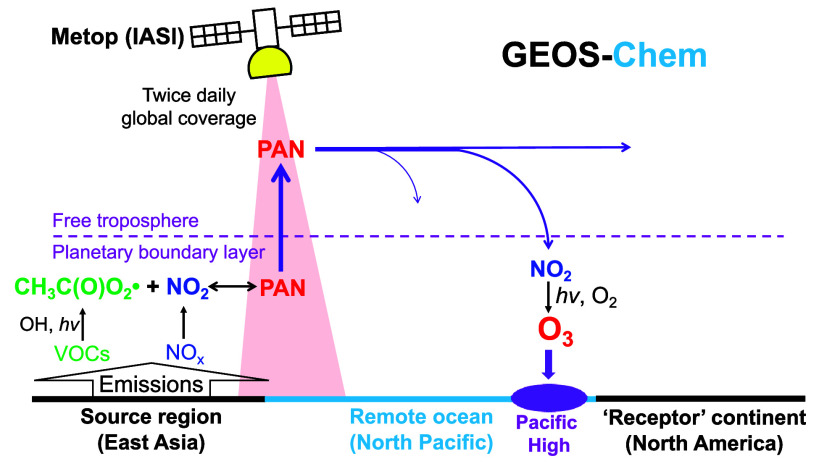

Peroxyacetyl nitrate (PAN) is produced in the atmosphere by photochemical oxidation of non-methane volatile organic compounds in the presence of nitrogen oxides (NO_*x*_), and it can be transported over long distances at cold temperatures before decomposing thermally to release NO_*x*_ in the remote troposphere. It is both a tracer and a precursor for transpacific ozone pollution transported from East Asia to North America. Here, we directly demonstrate this transport with PAN satellite observations from the infrared atmospheric sounding interferometer (IASI). We reprocess the IASI PAN retrievals by replacing the constant prior vertical profile with vertical shape factors from the GEOS-Chem model that capture the contrasting shapes observed from aircraft over South Korea (KORUS-AQ) and the North Pacific (ATom). The reprocessed IASI PAN observations show maximum transpacific transport of East Asian pollution in spring, with events over the Northeast Pacific offshore from the Western US associated in GEOS-Chem with elevated ozone in the lower free troposphere. However, these events increase surface ozone in the US by less than 1 ppbv because the East Asian pollution mainly remains offshore as it circulates the Pacific High.

## Abstract 2

This study presents the first satellite observations of transpacific transport of peroxyacetyl nitrate, a proxy and precursor of ozone pollution.

## 1 Introduction

Transpacific transport of Asian air pollution increases background surface ozone over the Western United States (US), making it more difficult to meet ozone air quality standards.^1−6^ This Asian influence has mainly been inferred from models but has been elusive to detect in observations.^1^ Observational studies of transpacific pollution generally use in situ and satellite measurements of carbon monoxide (CO) as a long-lived tracer of combustion influence,^4,7−10^ but elevated CO is not necessarily associated with ozone pollution. Here, we show that continuous infrared atmospheric sounding interferometer (IASI) satellite observations of peroxyacetyl nitrate (PAN),^11^ a long-lived photochemical tracer closely associated with ozone formation, provide a robust indication of transpacific ozone pollution.

PAN is produced together with ozone by photochemical oxidation of non-methane volatile organic compounds (NMVOCs) in the presence of nitrogen oxides (NO_*x*_).^12^ It is thermally unstable, with a lifetime of only 1 h at 295 K but doubling for every 4 K decrease in temperature to reach over 1 month in the mid-troposphere.^13^ It provides a reservoir for the long-range transport of NO_*x*_ from source regions to the remote atmosphere. PAN formed over East Asia in the planetary boundary layer (PBL) and ventilated to the cold free troposphere (FT) can be transported across the North Pacific before it subsides to release NO_*x*_.^9,14^ Aircraft observations off the US west coast show that PAN in descending air on the east branch of the semipermanent Pacific High decomposes and promotes efficient formation of ozone in the lower FT at 2–5 km altitude.^4,7,8,15^ This elevated lower FT ozone could then affect surface ozone air quality over the Western US by vertical mixing.^16,17^ Both aircraft measurements and model results show that PAN contributes significantly to transpacific ozone air pollution, adding to the directly transported ozone produced over East Asia.^4,8,18,19^

Despite the observation of lower FT ozone plumes off the US west coast, assessments of Asian pollution contribution to Western US surface ozone have been inconclusive. The elevated FT ozone transported across the Pacific could reflect the mixing of Asian pollution and stratospheric contributions.^16,20^ At US surface sites, the detection of Asian pollution ozone plumes has been difficult due to dilution during entrainment and other sources of ozone variability.^16,21,22^ At Western US high-altitude sites, although ozone filaments with concentrations enhanced by up to about 14 ppbv are observed, it is difficult to attribute their sources.^10,23^ Models have difficulty in resolving the transport of pollution plumes across the Pacific because of numerical diffusion under stretched-flow conditions.^24,25^ On the other hand, Asian PAN plumes can be distinctly detected at Western US high-altitude sites,^14,18^ suggesting that PAN observations by satellite could be useful for documenting transpacific transport of ozone pollution.

PAN is detectable from space in the thermal infrared (TIR). Early observations from the tropospheric emission spectrometer (TES) captured plumes associated with boreal wildfires^26,27^ but were too sparse to detect variability over the Pacific.^28^ More recent observations from the cross-track infrared sounder (CrIS) have detected plumes from wildfires and metropolitan areas.^29,30^ The IASI dataset is unique in its coverage and length, providing continuous global twice-daily mapping since 2007,^11,31^ and has shown consistency with ground-based PAN column measurements at remote sites.^32^

## 2 Materials and Methods

### 2.1 IASI PAN Observations

We use PAN column observations retrieved by version 4 of the artificial neural network framework for the IASI (ANNI).^11,31,33^ The IASI operates on the Metop series of polar-orbiting meteorological satellites and has a twice-daily global coverage (∼9:30 every morning and evening) with an elliptical footprint of 12 × 12 km^2^ at the nadir. The Metop series starts with Metop-A (launched on 19 October 2006 and retired on 30 November 2021) and goes on with Metop-B (launched on 17 September 2012) and Metop-C (launched on 7 November 2018). The ANNI provides a continuous record of PAN columns starting from October 2007. Here, we use the morning data averaged from Metop-A and Metop-B. The data are highly consistent between the two instruments (Figure S1).

The ANNI retrieval extracts the hyperspectral range index (HRI) as the PAN spectral enhancement above the background in the IASI 760–880 cm^–1^ spectrum.^11^ Although there are overlaps between the spectral signature of PAN and the other peroxyacyl nitrates (PANs), PAN accounts for at least 80% of the total PANs under different conditions.^34−36^ Therefore, IASI retrieval represents PAN for most conditions. A neural network is then used to convert this HRI into a column density [molecules cm^–2^]. The background is set by IASI spectra in the remote troposphere, with an assumed background PAN column density of 1.9 × 10^15^ molecules cm^–2^ from the ECHAM5/MESSy Atmospheric Chemistry (EMAC) model, and is added to the HRI-retrieved column density.^11,37^ Retrieved PAN can be lower than this background if the HRI is negative. The column retrieval of PAN is sensitive to the temperature at which PAN is located and, therefore, is sensitive to the assumed PAN vertical distribution. For its baseline retrieval, the ANNI assumes a constant vertical profile shape of PAN based on mean values from the EMAC model.^37^ This can be a large source of retrieval error because of the large variability in that shape.^12^

Here, we reprocess the IASI retrieval with local PAN vertical profile shapes from the GEOS-Chem chemical transport model by making use of the averaging kernels that are retrieved alongside the total column in the ANNI v4 algorithm.^33^ Specifically, the following equation is applied, which effectively replaces the constant a priori profile with GEOS-Chem vertical profiles^33^
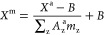
1where *X*^m^ is the column retrieved with the updated prior vertical profile (here from GEOS-Chem), *X*^a^ is the baseline column retrieved with the EMAC prior profile, and *B* = 1.9 × 10^15^ molecules cm^–2^ is the background column. The retrieval is done on a 14-level vertical grid, where *A*_z_^a^ is the averaging kernel describing the sensitivity of the retrieval to PAN at altitude z, and *m*_z_ is the normalized prior vertical profile defining the profile shape
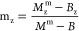
2Here, *M*^m^ is the total column from GEOS-Chem, *M*_z_^m^ is the partial column for the corresponding level, and *B*_z_ is the background vertical profile.^11^ After applying the averaging kernel, the retrieval postfilter needs to be reapplied,^33^ which means that we remove observations that do not meet criterion (1) or that meet both criteria (2) and (3) following Franco et al.^11^
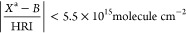
Criterion 1
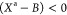
Criterion 2
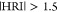
Criterion 3

In the following analysis, we grid IASI pixel data to the 4 × 5° GEOS-Chem horizontal grid to compare them with GEOS-Chem model results.

### 2.2 GEOS-Chem Model

We use GEOS-Chem version 13.4.1 (https://zenodo.org/records/6564702) with updates described below. GEOS-Chem is driven by meteorological data from the NASA Modern-Era Retrospective Analysis for Research and Applications, Version 2 (MERRA-2). We conduct global model simulations at a horizontal resolution of 4 × 5° with 72 vertical levels. A finer horizontal resolution is not used here because the accuracy of free tropospheric transport is limited by the model’s vertical rather than horizontal resolution,^25^ and 4 × 5° horizontal resolution is sufficient for simulating regional-scale photochemistry.^38^ Meanwhile, the KORUS-AQ PAN vertical profile from the 4 × 5° simulation is consistent with the 0.5 × 0.625° model results from our previous study.^39^ Emissions in GEOS-Chem are prepared by Harmonized Emissions Component (HEMCO).^40,41^ Global anthropogenic emissions are from the Community Emissions Data System (CEDSv2),^42^ superseded over China by the Multiresolution Emission Inventory (MEIC).^43,44^ We add ethanol emissions from seawater and transportation following Bates et al.^45^ Other emissions include NO_*x*_ from lightning^46^ and soil,^47^ MEGANv2 biogenic VOCs,^48^ dust,^49^ sea salt,^50^ and GFEDv4 open-fire emissions.^51^ Following Fischer et al.,^12^ we distribute 35% of the open-fire emissions by mass in the FT and partition, respectively, 40 and 20% of the open-fire NO_*x*_ emissions directly to PAN and HNO_3_.

We implement in our simulation particulate nitrate photolysis following Shah et al.^38^ Shah et al.^38^ show that including photolysis of particulate nitrate on sea salt aerosols can account for the missing NO_*x*_ source over the oceans in ATom aircraft observations, while Colombi et al.^52^ show that it largely corrects a negative ozone bias against ozonesonde observations over East Asia. Here, we see an increase in PAN from nitrate photolysis, which is an important factor for GEOS-Chem to reproduce IASI PAN (Section 3.2; Figure S2). We also adopt a slower peroxyacetic acid (PAA) + OH reaction rate of 3 × 10^–14^ cm^3^ molecules^–1^ s^–1^ as measured by Berasategui et al.^53^ and implemented in the latest GEOS-Chem model version 14.3.0.^54^ This rate is 40 times lower than the previously recommended value, but we find that this has only a minor effect on simulated PAN.

## 3 Results and Discussion

### 3.1 Vertical Profiles of PAN over South Korea and the Northeast Pacific

**Figure 1:**
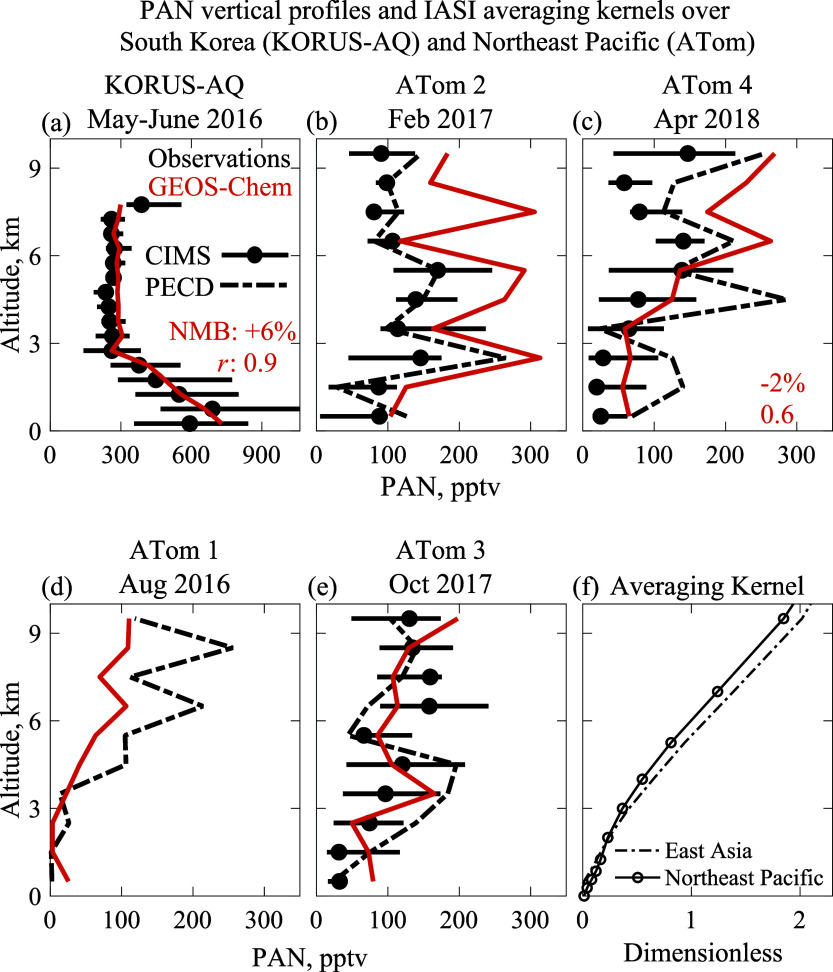
Vertical profiles of peroxyacetyl nitrate (PAN) concentrations in South Korea and the Northeast Pacific. Median observations from (a) KORUS-AQ aircraft campaign over South Korea and nearby waters (May–June 2016) and from (b–e) ATom aircraft campaign deployments over the Northeast Pacific (Figure S3) (15-55° N, 180–145° W) in different seasons of 2016–2018 are compared to the GEOS-Chem model sampled along the aircraft tracks. The KORUS-AQ measurements were made by the Georgia Tech chemical ionization mass spectrometer (GT-CIMS).^60,61^ The ATom payload included two PAN measurements, the GT-CIMS and the PANTHER (PAN and trace hydrohalocarbon experiment) gas chromatograph electron capture detector (PECD).^62^ The GT-CIMS was not included in the summer 2016 deployment.^63^ Horizontal bars indicate 25th–75th percentiles in the GT-CIMS observations. Normalized mean bias (NMB) and correlation coefficient (*r*) between observations (GT-CIMS for KORUS-AQ and PECD for ATom 4) and GEOS-Chem for the spring profiles are shown in the inset in (a, c). (f) IASI PAN averaging kernels over East Asia and the Northeast Pacific, respectively, averaged over the KORUS-AQ and ATom flight track domains for May 2016.

Figure 1a–e show the vertical profiles of PAN measured from aircraft over South Korea in spring during the KORUS-AQ campaign^55,56^ and over the Northeast Pacific in different seasons during the four ATom campaign deployments (Figure S3),^57,58^ compared to the GEOS-Chem vertical profiles sampled along the flight tracks. KORUS-AQ and ATom show contrasting vertical profiles over the East Asia source region and the remote North Pacific. PAN in KORUS-AQ is enhanced in the PBL with a concentration of 600–700 pptv at 0–1 km decreasing with altitude, flattening to a uniform concentration of 270 pptv in the FT above 3 km altitude. This is closely reproduced by GEOS-Chem, where the PBL enhancement is driven by East Asian anthropogenic emissions. The vertical profile shapes are reversed over the North Pacific, with minimum concentrations in the marine boundary layer (MBL) and increasing concentrations in the FT above. Such vertical profiles of PAN over the North Pacific are expected from the cold reservoir aloft and thermal decomposition during subsidence.^12^ There is seasonal variation in the FT vertical profile as expected from different lifting altitudes for continental pollution transported to the Pacific with maxima in the lower FT in the winter, in the middle troposphere in the spring and autumn, and in the upper troposphere in the summer. The vertical profiles and their seasonality are again well captured by GEOS-Chem, with the best model performance in the spring that is most pertinent to this study. Although the model overestimates PAN in the middle and upper FT in winter, it performs reasonably well in the PBL and lower FT where concentrations are high. The underestimate in summer could be due to an underestimate of PAN production in open-fire plumes^12^ and biased low injection heights of fire emissions.^59^

The vertical profile of averaging kernels *A_z_*^a^ (eq 1) describes the sensitivity of the satellite retrievals to concentrations as a function of altitude. Here, we see an order of magnitude increase in IASI PAN *A*_*z*_^a^ from the PBL to the FT for the KORUS-AQ and ATom conditions (Figure 1f). This is a critical issue for PAN retrieval, considering the systematic variability of the PAN vertical profile shapes illustrated in Figure 1a–e. Assuming a single profile globally, as in the baseline IASI retrieval, can induce large errors. The success of GEOS-Chem in reproducing the observed variability in the vertical profile shape indicates that the baseline IASI retrieval can be reprocessed with the local normalized GEOS-Chem profiles as prior information, following the method described in Section 2.1. This reprocessing is also necessary for comparing GEOS-Chem to the IASI column concentrations.

### 3.2 Transpacific Transport of PAN Observed by the IASI

**Figure 2:**
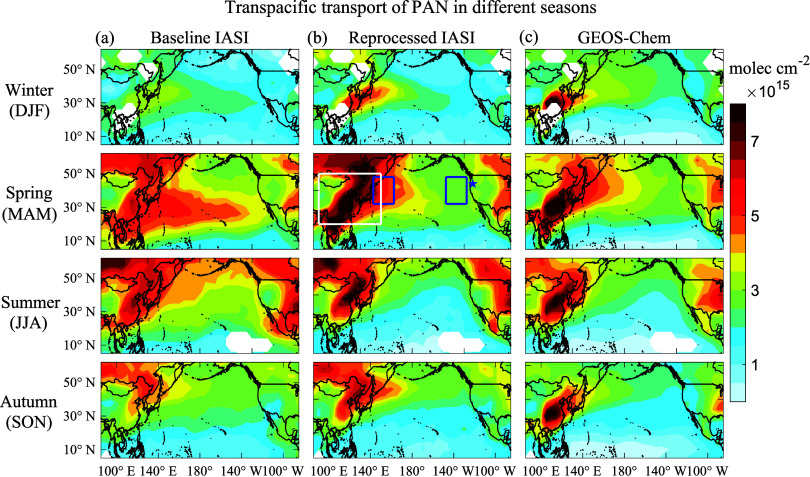
PAN column densities across the Pacific region in different seasons. Seasonal mean (a) baseline and (b) reprocessed IASI satellite observations for 2016 are compared to (c) GEOS-Chem model sampled at the locations and times of valid IASI observations. The results shown are daytime averages for Metop-A and Metop-B observations. White areas have fewer than 40% valid retrievals. The baseline IASI retrieval assumes a global mean normalized vertical profile from the EMAC model. The reprocessed IASI retrieval uses local normalized vertical profiles from GEOS-Chem, thus accounting for very different vertical shapes over different regions. Rectangles denote the Northwest Pacific (32–48° N, 142.5–162.5° E), Northeast Pacific (32–48°N, 147.5–127.5° W), and East Asia (20–50° N, 100–150° E) regions used in the analysis of Section 3.3. The blue star is the location of the Mt. Bachelor Observatory (MBO) site (44.0° N, 121.7° W; 2.74 km asl).

Figure 2a,b compare the baseline IASI retrieval over East Asia and the North Pacific to the reprocessed retrieval using local GEOS-Chem normalized vertical profiles for the year 2016. The reprocessed retrieval increases PAN over source regions and immediately downwind (where PAN peaks at low altitudes) and decreases PAN in the nonwinter remote atmosphere (where PAN peaks at high altitudes). The seasonal maximum over East Asia shifts from summer to spring. There is also a poleward shift because PAN at higher latitudes tends to be present at low altitudes due to colder surface temperatures.^12^ Column PAN as measured by the IASI is most sensitive to the FT and so would have relatively little diurnal variation, even over source regions. We used the reprocessed PAN retrievals for further analysis.

We see from the reprocessed PAN retrievals in Figure 2b that PAN over East Asia peaks in the spring, reflecting a combination of active photochemistry and low temperatures. This is also the season when the Asian outflow to the Pacific is the strongest,^64^ stretching longitudinally across the Pacific. In the summer and autumn, the Asian outflow is shifted to higher latitudes. The wintertime outflow is limited by the weak source of PAN and the suppressed lifting. Figure 2c shows the GEOS-Chem PAN columns sampled at the locations of valid IASI retrievals. GEOS-Chem reproduces closely the reprocessed IASI observations over East Asia and across the Pacific, including the seasonality. It underestimates PAN at high latitudes over Russia and Canada, possibly due to the underestimate of PAN production in open-fire plumes.^12^

### 3.3 Transpacific PAN Events and Implications for Transpacific Ozone Pollution

**Figure 3:**
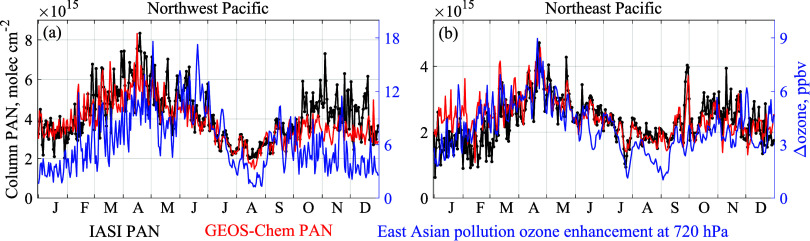
Daily time series of PAN column densities averaged over (a) Northwest and (b) Northeast Pacific (blue rectangles in Figure 2b) from the reprocessed IASI PAN observations and from the concurrent GEOS-Chem simulation in 2016. The results shown are daytime averages for Metop-A and Metop-B observations. The evening data are highly consistent with the morning data over the North Pacific, with half-day deviations as compared with morning data for some NE Pacific events (Figure S5). Also shown are the Asian pollution enhancements of 720 hPa ozone concentrations in GEOS-Chem as diagnosed by the difference with a sensitivity simulation that zeros anthropogenic emissions in the large white rectangle of Figure 2b.

Figure 3 shows the full-year IASI and GEOS-Chem time series of daily PAN column concentrations over the NW and NE Pacific regions (blue rectangles in Figure 2b) most relevant for the transpacific transport of Asian pollution to the US. Also shown in Figure 3 are the East Asian anthropogenic pollution enhancements of ozone concentrations in the lower free troposphere at 720 hPa (≈3 km altitude) in GEOS-Chem, as computed by difference with a sensitivity simulation in which anthropogenic NO_*x*_, NMVOC, and CO emissions over East Asia (large white rectangle in Figure 2b) are set to zero except for airplanes, ships, and fertilizer-driven soil emissions. We focus on ozone enhancement at the lower free troposphere because it controls the surface ozone background.^17^ Ozone at 720 hPa is representative of the 2–5 km altitude range (Figure S4). PAN peaks in April over both the NW and NE Pacific and has a secondary maximum in autumn, consistent with the meteorological seasonality of the Asian outflow to the Pacific.^64^ GEOS-Chem reproduces the observations closely except for a 20% underestimate over the NW Pacific from October to November and a 40% overestimate over the NE Pacific in January. Monthly IASI PAN variations are reproduced by the GEOS-Chem model with correlation coefficients, respectively, of 0.92 and 0.69 over the NW and NE Pacific. Day-to-day variability including events of Asian outflow and transpacific transport is also captured by GEOS-Chem, with deseasonalized correlation coefficients of 0.42 over the NW Pacific and 0.58 over the NE Pacific. In the GEOS-Chem model, the East Asian pollution PAN enhancement (PAN produced by East Asian anthropogenic emissions as represented in the sensitivity simulation) is strongly correlated with total PAN in the daily time series over the NW and NE Pacific, with a correlation coefficient, respectively, of 0.81 and 0.78, indicating that East Asian pollution effectively drives high-PAN events.

Asian pollution influence on ozone over the Western US is known from observations and models to peak in April–May^3,65−68^ and this is apparent in the IASI PAN observations. Figure 3 shows that the Asian pollution enhancement of ozone over the NW and NE Pacific as simulated by GEOS-Chem closely tracks IASI PAN, peaking in April, indicating that IASI PAN can serve as a tracer for ozone pollution. The Asian pollution enhancement of ozone over the NW Pacific shows a second peak in June, due to direct transport of ozone during the ozone peak season (May–July) in East Asia.^52^ There is no associated ozone enhancement over the NE Pacific because transport in the summer is shifted to higher latitudes (Figure 2). Although ozone observations are also available from the IASI, they have too little sensitivity to the lower troposphere.^69^ The observed PAN is a better indicator of Asian ozone pollution.

**Figure 4:**
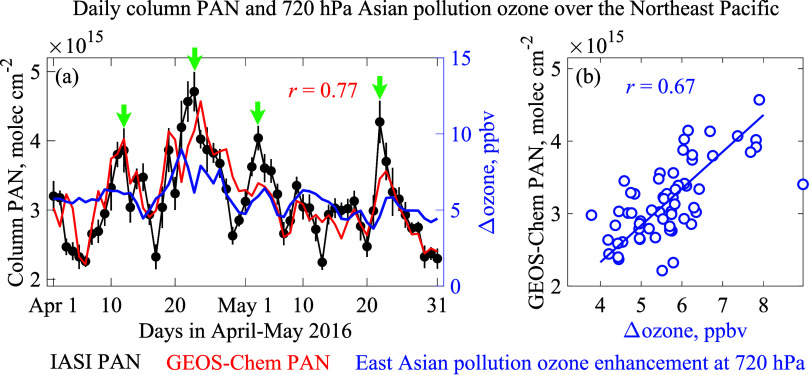
Daily PAN column densities and relation to Asian ozone pollution enhancements at 720 hPa averaged over the Northeast Pacific during April–May 2016. (a) Time series is an excerpt from Figure 3b. Arrows indicate the PAN peaks in the IASI data. The vertical bars are standard errors (SEs) on those observed averages. (b) Scatterplot shows the daily correlation between PAN and Asian ozone pollution enhancements in the GEOS-Chem model. The inset in the left panel is the correlation coefficient (*r*) between the IASI and GEOS-Chem PAN, and the inset in the right panel is that between PAN and 720 hPa East Asian pollution ozone enhancement in GEOS-Chem.

Figure 4a zooms in on the April–May 2016 period of Figure 3 time series over the NE Pacific. There are four PAN peaks (April 12, April 23, May 3, and May 22), and GEOS-Chem captures them all with a day-to-day correlation coefficient of 0.77. The episodic nature of transpacific pollution events is well known, driven by frontal lifting over the Asian continent and the position of the North Pacific High.^4,64^ We conducted GEOS-Chem sensitivity simulations zeroing out separately East Asian anthropogenic emissions, open-fire emissions, and Southeast Asia biogenic VOC emissions.^70^ We find that the high-PAN events during April–May 2016 over the NE Pacific are mainly from East Asian anthropogenic enhancements, except for the May 22–24 event where fires are also important. Open fires in Russia could dominate the transpacific transport of PAN in some years.^18^ We find that Southeast Asia makes little contribution to the events.

High-PAN events in GEOS-Chem over the NE Pacific are associated with East Asian pollution ozone enhancements at 720 hPa (Figure 4b). The scatterplot shows the relationship between total PAN columns and Asian ozone pollution enhancements in the model. The strong correlation (*r* = 0.67) implies that IASI observations of high-PAN events can be used as a proxy for events of Asian ozone pollution transported across the Pacific. The dynamic range for Asian ozone pollution in the model is relatively small, with a background of 5 ppbv and events peaking at 9 ppbv. Observations of Asian pollution plumes in the lower FT over the NE Pacific indicate ozone enhancements of over 40 ppb.^7^ The weaker enhancements in the model likely reflect the numerical diffusion of Asian plumes during stretched-flow transport across the Pacific.^24,25^

**Figure 5:**
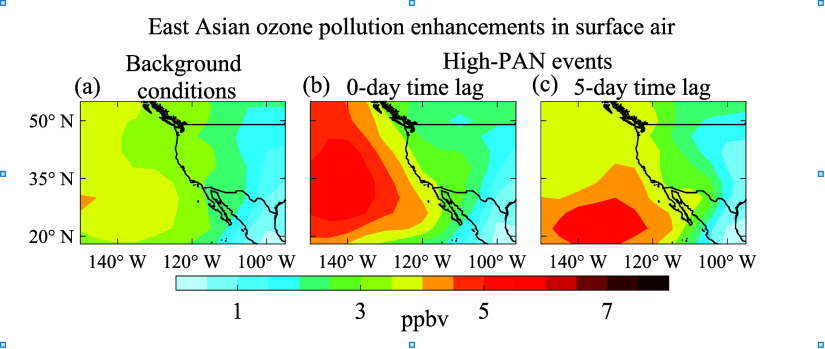
East Asian ozone pollution enhancements in surface air in April–May 2016 were associated with transpacific PAN transport events. Ozone enhancements under (a) background conditions and (b) during high-PAN events (Figure 4a) and (c) with a 5-day time lag. The East Asian ozone pollution enhancements are diagnosed in GEOS-Chem with a sensitivity simulation shutting off East Asian anthropogenic emissions.

Finally, we link the transpacific transport of PAN to East Asian pollution ozone enhancements in Western US surface air in April–May as diagnosed by GEOS-Chem (Figure 5). Here, we define high-PAN events in the model as April 10–12, April 21–26, May 2–5, and May 22–24, covering the four PAN peaks identified in Figure 4a. PAN during those events averages 3.9 × 10^15^ molecules cm^–2^, 35% higher than the background conditions (defined as periods outside of the high-PAN events) when PAN averages 2.9 × 10^15^ molecules cm^–2^. We find that Asian pollution ozone enhancements in surface air over the Western US are not significantly elevated during these high-PAN events, at most by 1 ppbv on top of the background Asian pollution enhancement of about 3 ppbv that reflects hemispheric-scale pollution rather than direct transpacific transport.^71^ Adding time lags for subsidence of high-PAN pollution events to the surface does not change this picture, as illustrated in Figure 5c with a 5-day time lag. Most of the Asian ozone pollution remains offshore and circulates around the North Pacific High as it subsides, skirting the US and eventually being entrained in the tropical easterlies. Such a circulation for transpacific pollution has been shown in previous studies.^4,8^ Dilution during boundary layer entrainment and mixing further reduces the signature of Asian pollution in the surface air. Even at the Mt. Bachelor Observatory (MBO) site (2.8 km asl; location shown in Figure 2b) under direct FT influence, ozone enhancements in Asian pollution plumes are usually too weak to observe.^4^ In contrast, PAN enhancements are readily observable.^18^ No PAN observations are available for MBO in spring 2016, but comparison to the Fischer et al.^18^ observations in spring 2008 shows consistency with transpacific PAN events observed by the IASI (Figure S6).

In summary, we have shown that IASI satellite observations of PAN across the North Pacific provide a proxy for the transpacific transport of Asian ozone pollution. We reprocessed the IASI PAN product to use normalized vertical profiles of PAN concentrations from the GEOS-Chem chemical transport model as prior information after showing that GEOS-Chem can reproduce the contrasting vertical profiles observed from the aircraft over East Asia and over the North Pacific in different seasons. Transpacific transport of PAN observed by the IASI is strongest in spring, with a secondary maximum in autumn, and is highly correlated in GEOS-Chem with the transpacific transport of Asian ozone pollution. Distinct high-PAN events of Asian pollution origin are observed over the Northeast Pacific in spring and are associated with ozone enhancements in the lower free troposphere, but the impact of these events on surface ozone in the US is insignificant because most of the Asian ozone pollution remains offshore in the circulation around the North Pacific High.

## Supporting Information Available

Comparisons of Metop-A and Metop-B observations; impact of nitrate photolysis on the GEOS-Chem model simulation of PAN column densities; ATom flight tracks over the Northeast Pacific; East Asian pollution ozone enhancement over the Northwest and Northeast Pacific at different altitudes; comparisons of morning and evening IASI PAN over the Northwest and Northeast Pacific; and daily variations of PAN from the IASI and in situ measurements at the Mt. Bachelor Observatory (PDF)
