# Supplementary material for: Transpacific Transport of Asian Peroxyacetyl Nitrate (PAN) Observed from Satellite: Implications for Ozone
Source: Environ Sci Technol. 2024 May 22;58(22):9760–9. doi: 10.1021/acs.est.4c01980 (PMC11155249; doi:10.1021/acs.est.4c01980)
Supplement: Supplementary file 1 — es4c01980_si_001.pdf [file es4c01980_si_001.pdf]

Supporting Information for

Transpacific transport of Asian peroxyacetyl nitrate (PAN) observed from satellite: implications for ozone

Shixian Zhai[1,2,], Daniel J. Jacob[2], Bruno Franco[3], Lieven Clarisse[3], Pierre Coheur[3], Viral Shah[2,#], Kelvin H. Bates[2,4], Haipeng Lin[2], Ruijun Dang[2], Melissa P. Sulprizio[2], L. Gregory Huey[5], Fred L. Moore[6,7], Daniel A. Jaffe[8,9], Hong Liao[10]*

[1] Earth and Environmental Sciences Programme and Graduation Division of Earth and Atmospheric Sciences, Faculty of Science, The Chinese University of Hong Kong, Sha Tin, Hong Kong SAR, China

[2] John A. Paulson School of Engineering and Applied Sciences, Harvard University, Cambridge, MA 02138, USA

[3] Université libre de Bruxelles (ULB), Spectroscopy, Quantum Chemistry and Atmospheric Remote Sensing, Brussels B-1050, Belgium

[4] NOAA Chemical Sciences Laboratory, Earth System Research Laboratories, & Cooperative Institute for Research in Environmental Sciences, University of Colorado, Boulder, CO 80305, USA

[5] School of Earth and Atmospheric Sciences, Georgia Institute of Technology, Atlanta, GA 30332, USA

[6] NOAA Global Monitoring Laboratory, Boulder, CO 80305, USA

[7] Cooperative Institute for Research in Environmental Sciences, University of Colorado Boulder, Boulder, CO 80309, USA

[8] School of Science, Technology, Engineering, and Mathematics, University of Washington, Bothell, WA 98011, USA

[9] Department of Atmospheric Sciences, University of Washington, Seattle, WA 98195, USA

[10] Jiangsu Key Laboratory of Atmospheric Environment Monitoring and Pollution Control, Collaborative Innovation Center of Atmospheric Environment and Equipment Technology, School of Environmental Science and Engineering, Nanjing University of Information Science and Technology, Nanjing 210044, China

[#] Now at Global Modeling and Assimilation Office (GMAO), NASA Goddard Space Flight Center, Greenbelt, MD 20770, USA and Science Systems and Applications, Inc., Lanham MD 20706, USA

Corresponding author: Shixian Zhai (shixianzhai@cuhk.edu.hk)

Supplementary Information:

Pages: S1-S8

Figures: S1-S6

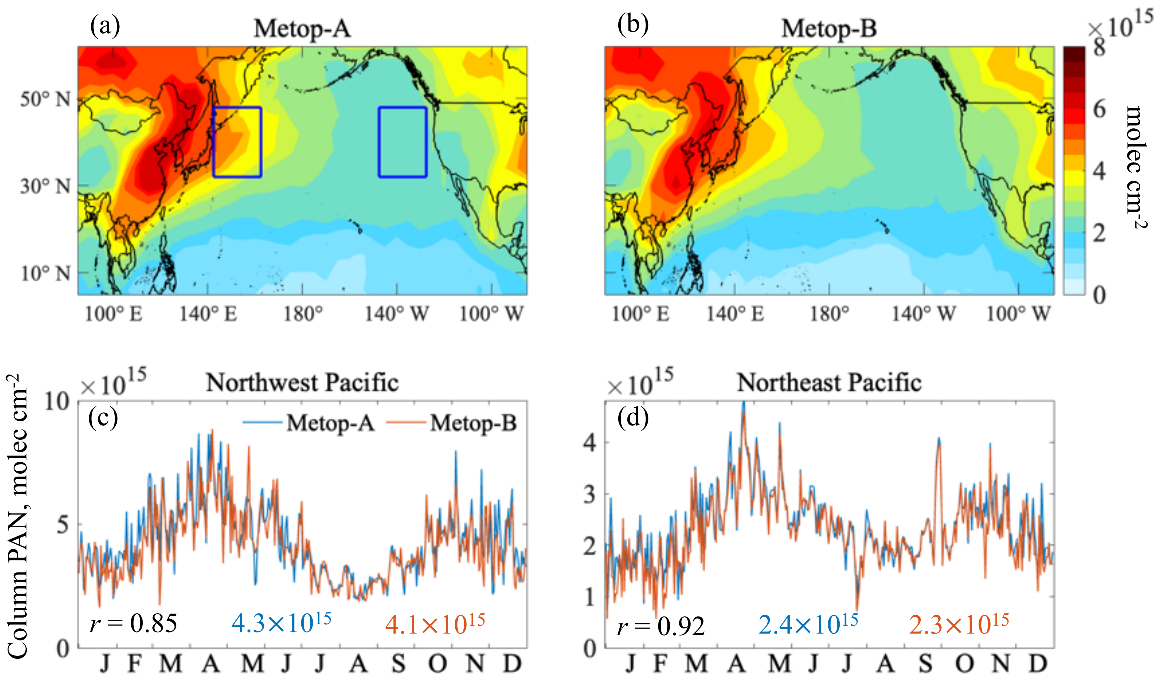

Figure S1. Comparisons of daytime PAN column densities from Metop-A and Metop-B for the year 2016. Panels (a) and (b) are the annual mean spatial distributions across the Pacific while panels (c) and (d) are daily time series over the Northwest and Northeast Pacific. Inset in panels (c) and (d) are the correlation coefficient (*r*) between the Metop-A and Metop-B daily series and their corresponding mean values.

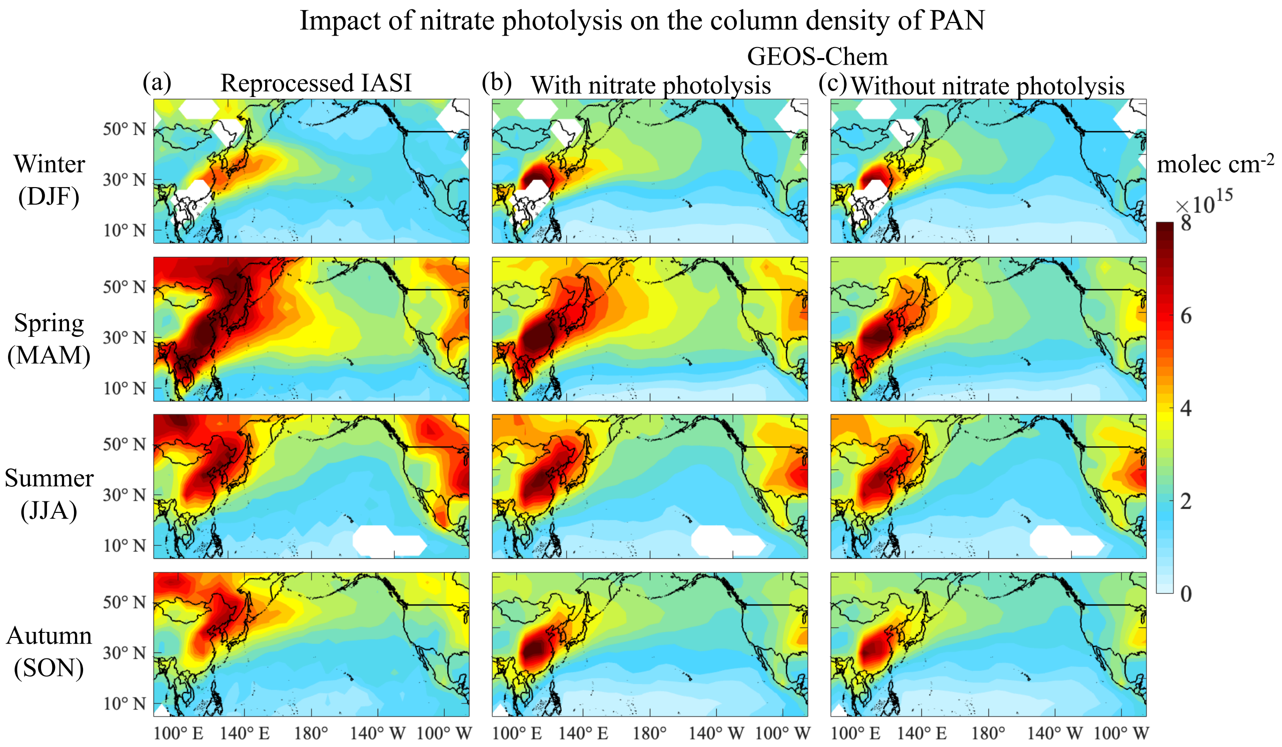

Figure S2. Seasonal mean PAN column densities across the Pacific from (a) IASI and GEOS-Chem (b) with and (c) without nitrate photolysis.[1]

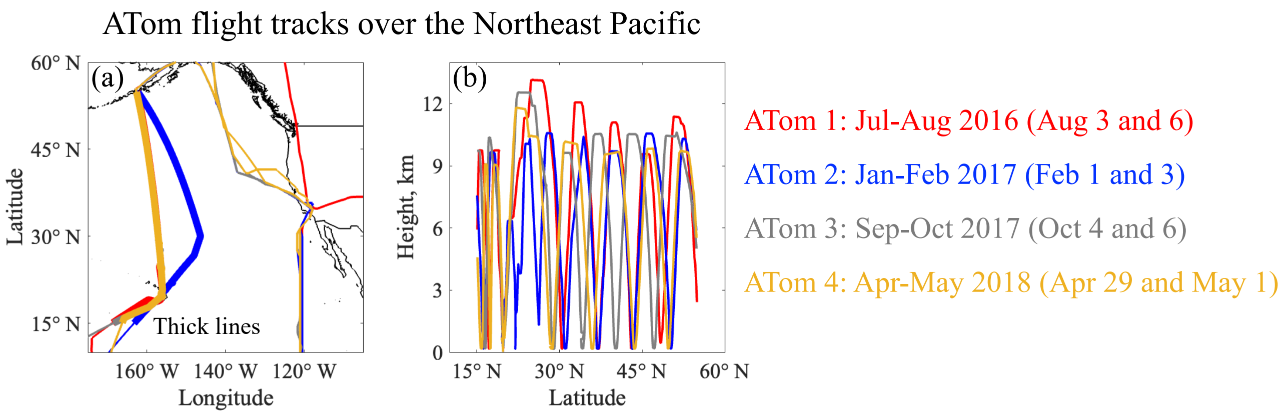

Figure S3. (a) Horizontal distributions and (b) latitude-height cross-sections of ATom flight tracks over the Northeast Pacific. Observations along the thick flight tracks in panel (a) are chosen in this study with the corresponding chosen flight dates shown in the brackets to the right of panel (b). ATom-1, ATom-3, and ATom-4 flight tracks are on top of each other in panel (a).

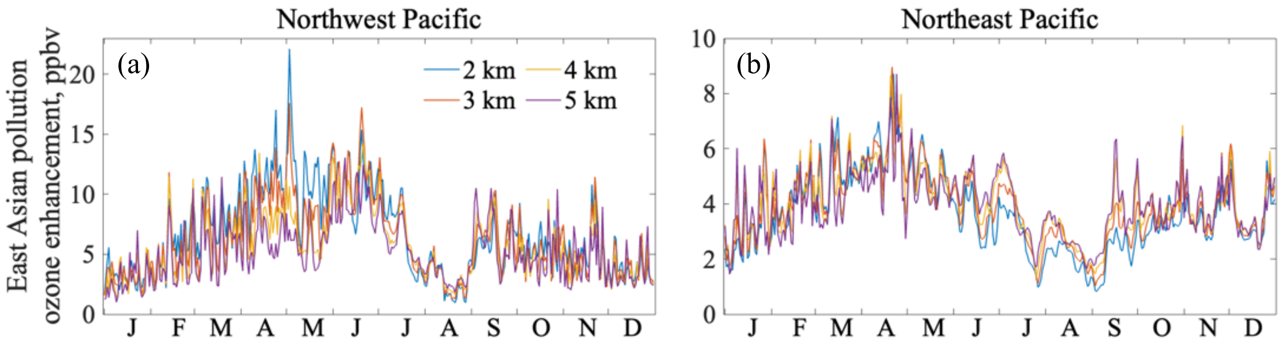

Figure S4. Daily time series of East Asian pollution ozone enhancement over the Northwest and Northeast Pacific regions at altitudes of 2 km, 3 km, 4 km, and 5 km from the GEOS-Chem model.

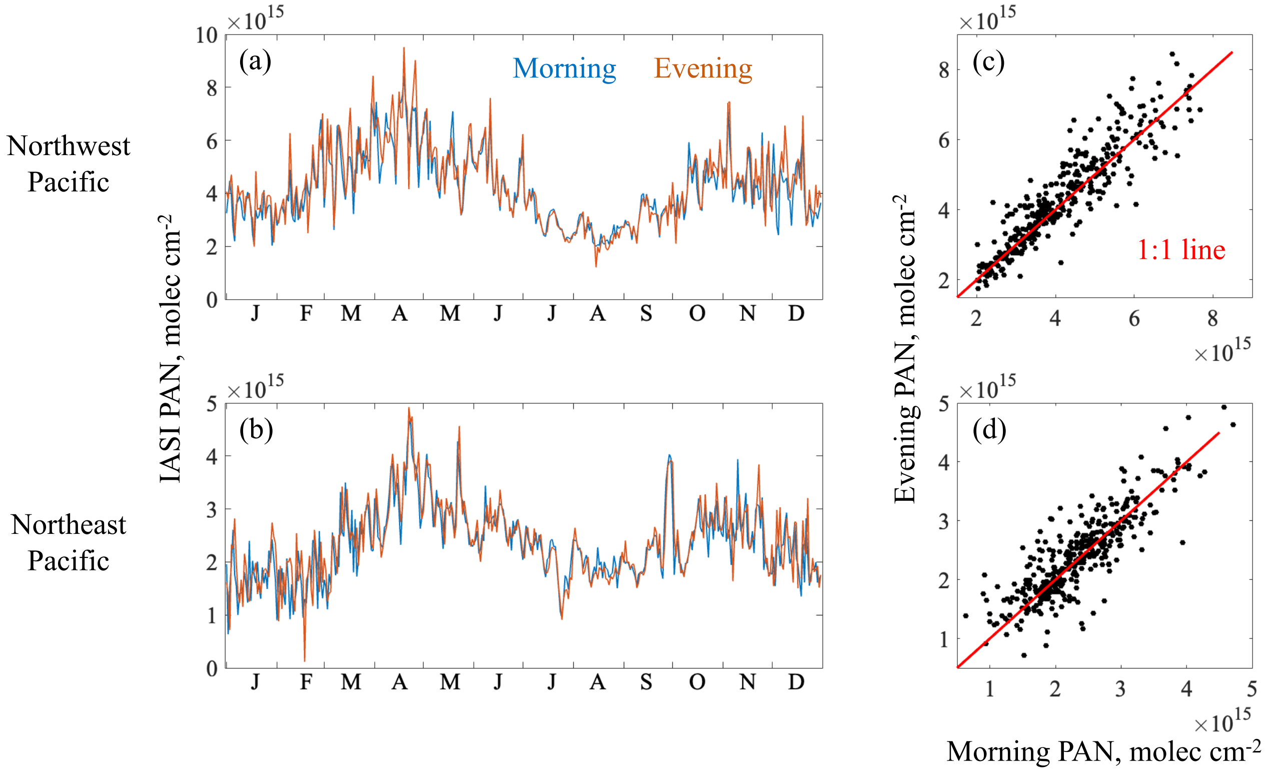

Figure S5. Comparisons of daily morning and evening IASI PAN over the Northwest and Northeast Pacific regions. Panels (a) and (b) are daily time series while panels (c) and (d) are corresponding daily scatter plots.

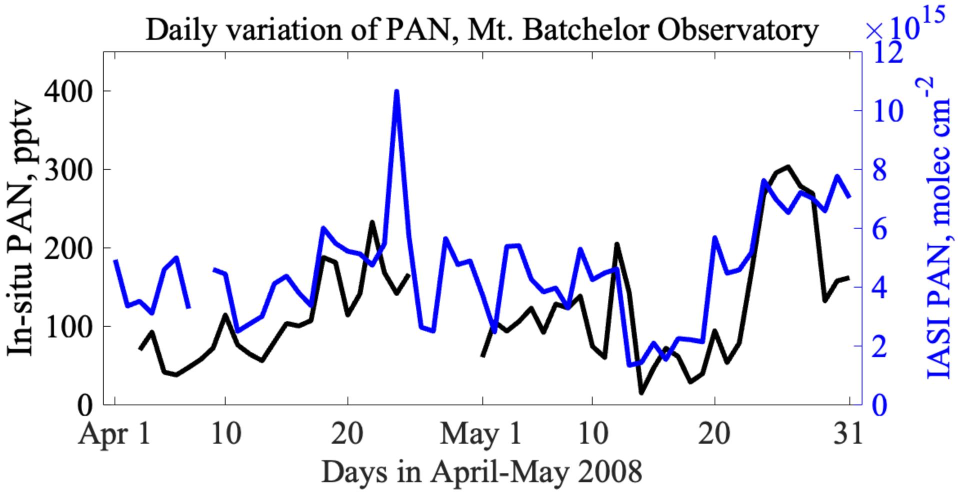

Figure S6. Consistent daily temporal variations of PAN from IASI and in-situ measurements at the Mount Bachelor Observatory (43.979° N, 121.687° W; 2.7 km a.s.l.).

References

(1) Shah, V.; Jacob, D. J.; Dang, R.; Lamsal, L. N.; Strode, S. A.; Steenrod, S. D.; Boersma, K. F.; Eastham, S. D.; Fritz, T. M.; Thompson, C.; et al. Nitrogen oxides in the free troposphere: implications for tropospheric oxidants and the interpretation of satellite NO_2 measurements. *Atmos. Chem. Phys.* **2023**, *23* (2), 1227-1257. DOI: 10.5194/acp-23-1227-2023.
